# The impact of diabetes and obesity on the severity and mortality of SARS-CoV-2 infection

**DOI:** 10.1007/s40200-025-01706-5

**Published:** 2025-09-01

**Authors:** Tomasz Klaudel, Michał Pelczarski, Monika Zaborska, Jakub Sadowski, Samanta Anna Ostrowska, Adam Drzymała, Rafał Jakub Bułdak

**Affiliations:** 1 Student Scientific Society of Clinical Biochemistry and Regenerative Medicine, Department of Clinical Biochemistry and Laboratory Diagnostics, Institute of Medical Sciences, University of Opole, Oleska 48, Opole, 45-052 Poland; 2 Department of Clinical Biochemistry and Laboratory Diagnostics, Institute of Medical Sciences, University of Opole, Oleska 48, Opole, 45-052 Poland

**Keywords:** SARS-CoV-2, COVID-19, Obesity, Diabetes, Mortality

## Abstract

**Purpose of review:**

The purpose of the study was to collect and summarise information available in the scientific literature on the probable reasons that lead to negative outcomes of COVID-19 in patients with pre-existing obesity and /or type 2 diabetes mellitus and influence on their treatment as also mortality.

**Recent findings:**

During the COVID-19 pandemic, it was observed that disease severity is often correlated with existing comorbidities, mainly in older obese male patients. SARS-CoV-2-infected patients with chronic diseases required hospitalisation more often and their overall prognosis is worse. The following review describes the impact of obesity and diabetes on the SARS-CoV-2 infection course and mortality risk.

**Summary:**

Diabetes and obesity have a multifactorial impact on the risk of SARS-CoV-2 virus infection, as well as on the nature and dynamics of the development of the infection. In turn, the presence of these diseases significantly increased the risk of requiring intensified treatment, complications and ultimately death. Limited access to medical care systems due to the pandemic and the impact on everyday activities made it even more difficult to control diabetes and obesity, leading to the deterioration of patient’s condition and the occurrence of new cases of disease. Therefore, it is necessary not only to appropriately modify treatment of those already infected, but also to use appropriate prevention to reduce the number of potential high-risk patients.

## Introduction

**Fig. 1:**
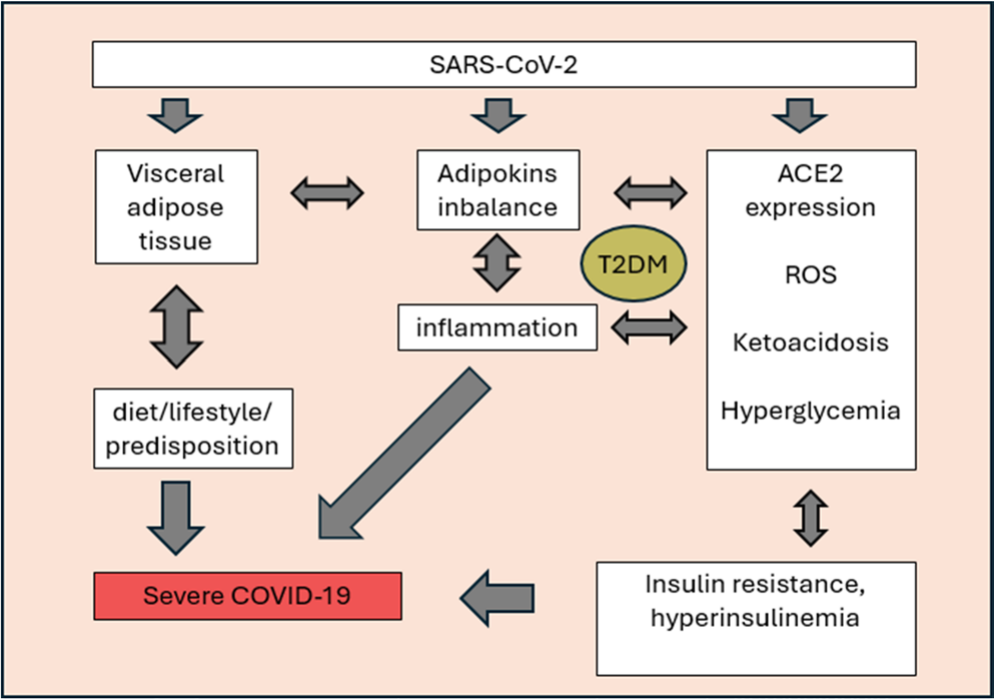
Simplified diagram of the relationship between obesity and diabetes and their impact on the severity of COVID-19. Visceral adipose tissue is responsible for proinflammatory secretion. It generates chronic systemic inflammation [12]. Hyperglycemia developed by diabetes is responsible for the occurrence of ketoacidosis incidents, higher ACE2 expression and increased ROS levels [13–16]. The effect of the coexistence of these terms in the case of SARS-CoV-2 infection is its more likely severe course [4, 17, 18]. Figure 1 was created using the Servier Medical Art Commons Attribution 4.0 Unported Licence http://smart.servier.com. https://creativecommons.org/licenses/by/4.0/

Obesity affects more than 1 billion people worldwide [1]. It has been shown that a *BMI* of at least 30.0 kg/m2 is most common in COVID-19 patients under 64 years of age. A trend of obesity prevalence has been observed in the younger population [2]. The incidence of this disease is increasing by 125% in girls and 100% in boys. This could lead to up to 400 million cases of childhood obesity in the next decade [3]. Obesity is considered the most important factor accelerating the development of type 2 diabetes and, as a consequence, these diseases often coexist [4]. Moreover, SARS-CoV-2 infection is also associated with inflammation, which can be enhanced by proinflammatory cytokines (adipokines) secreted by the adipose tissue of obese/diabetic patients (Fig. 1).

There are two main types of adipose tissue in the human body. Adipose tissue, as an “endocrine organ”, secretes pro- and anti-inflammatory adipokines from adipocytes, which activates the immune system. Abnormal accumulation of adipose tissue directs the AT secretory profile to the pro-inflammatory side in obese individuals [5]. Physical activity (PA) reduces the size of adipocytes and sets the AT secretory profile to the anti-inflammatory side. It is known that PA affects TNF-a, leptin, and also enhances lipolysis [6]. In the case of visceral obesity, adiponectin levels will be reduced, while increased levels of IL-6, TNF-α and CRP will be observed. An unfavorable lifestyle may be associated with a more severe SARS-CoV-2 infection and increased mortality. The presence of obesity through the development of insulin resistance promotes the development of diabetes. Metabolic disorders accompanying diabetes affect the risk of developing obesity. In both conditions, the imbalance between pro- and anti-inflammatory processes, accompanied by vascular changes, altered protein expression, and hyperglycemia itself, contributes to susceptibility to homeostasis disruption in the event of infection. Both obesity and diabetes significantly affect the course and prognosis of infectious diseases, often coexisting with each other. This paper presents a detailed description of the pathophysiology of the above-mentioned mechanisms and their connections (Fig. 1).

Perivascular adipose tissue (PVAT) has the ability to produce inflammatory cytokines and growth factors (GFs) [7]. It is considered to be a significant factor contributing to the development of vascular changes. Long-term inflammation leads to organ damage through immune system activation [8]. These processes translate into a gradual loss of pancreatic B cell function, which is involved in the development of type 2 diabetes. TNF-α is involved in this process. Its elevated levels resulting from obesity induce a decrease in the expression of glucose transporter 4 (GLUT4), resulting in impaired glucose uptake from the circulation into tissues [9]. TNF-α derived from brown adipocytes activates the MAPK (mitogen-activated protein kinase) signaling pathway, which prevents the formation of the normal insulin receptor. This process also occurs in IRS-1 (Insulin Receptor Substrate 1), which consequently leads to hyperglycemia induced by insulin resistance [10]. In patients with type 2 diabetes, serum TNF-α levels are higher than in the control group. The difference is also noticeable in the group of patients with type 2 diabetes and obesity compared to diabetics without excessive body weight (Fig. 1). Increased TNF-α concentration also correlates with increased HbA1C concentration in patients with type 2 diabetes, which indicates its significant role in the development of hyperglycemia [11].

In summary, excessive adipose tissue correlates with increased secretion of pro-inflammatory factors secreted by adipocytes. This weakens the immune system and translates into increased susceptibility to infections and the development of a “cytokine storm”. This correlation was noted in the works of Jiun-Ling Wang et al. [19], Xiude Fan et al. [20], Jaber Abdullah Alshahrani et al. [21].

A significant feature of the pandemic was the change in the perception of the greatest risk factor for COVID-19 patients from chronic obstructive pulmonary disease (COPD) to obesity, diabetes and related metabolic disorders. There were significant differences in both severity and mortality between control groups and groups with type 2 diabetes and obesity, confirmed by various studies. Differences were also noticeable among ICU (intensive care unit) patients. Before the pandemic, obese patients constituted 15–20% of all admissions. The pandemic caused a twofold increase (up to 40%) among them [22]. This indicates that both conditions are strong predictors of the outcome of COVID-19 infection (Table 2). A cohort study by Zhou et al. found that 31% of people who died from COVID-19 in China had diabetes [23]. The aim of this article is to discuss in detail some aspects of the above correlation.

## Search methodology

**Fig. 2:**
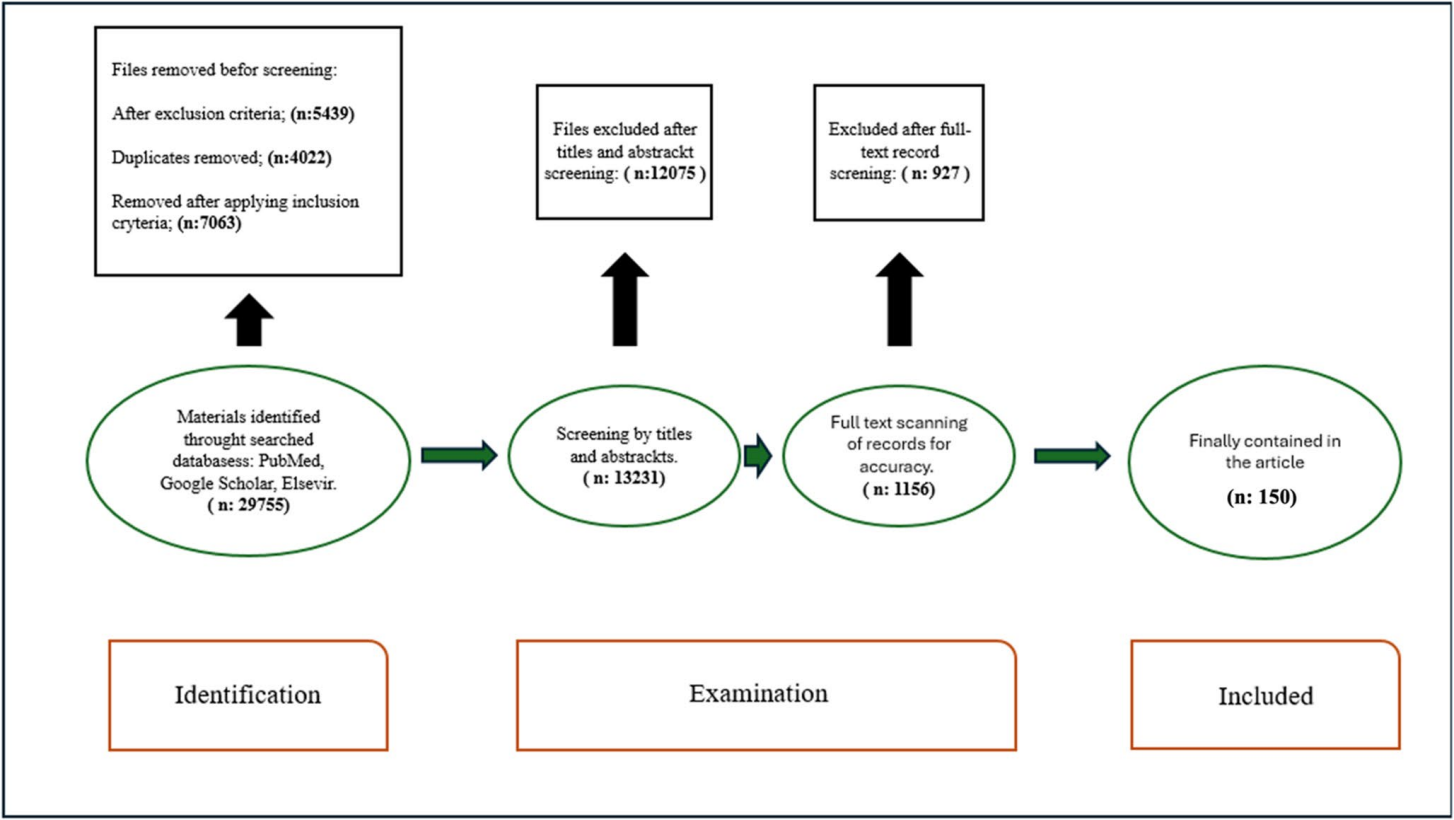
Search strategy for papers included in the manuscript

Figure 2 shows the authors’ search strategy. The search strategy was based on the PRISMA protocol, but it is a general overview. The numerical results of each search are presented, including inclusion and exclusion criteria. Literature review was conducted by each of the authors separately. Inclusion criteria for this review were established as: (1) original article, meta-analysis, general review, letter to the editor and case report, (2) online documents including books and guidelines, (3) sources referring to the course of COVID-19 infection in diabetic patients, (4) articles focusing on course of covid-19 infection in obese patients, (5) Sources treating the prognosis of COVID-19 patients under the influence of concomitant diseases, (6) articles and sources referring to selected keywords, focusing specifically on the pathophysiology, prognosis, treatment of the course of SARS-CoV-2 infection after the aforementioned conditions. The following exclusion criteria were used: (1) articles and sources that were found not scientific, anecdotal, or outdated, (2) articles published before the year 2015, excluding single sources retained due to their value for the review, (3) sources insufficiently focused on pathophysiology, prognosis and treatment in COVID-19 patients with diabetes, obesity and their combination. The final result was a comprehensive literature review, encompassing a total of 150 articles including research papers, scientific reviews, guidelines, case reports, meta-analyses, systematic reviews, and general reviews.

### SARS-CoV-2 infection

The most common symptoms of SARS-CoV-2 infection include cough, sore throat, back pain, fever, shortness of breath, chills, chest tightness, fatigue and weakness with more specific taste and smell disorders [24]. An increasing number of atypical symptoms have also been reported, such as stomach problems, hearing disorders, as well as the occurrence of dermatological changes, including skin rashes [25].

Widely accepted mechanism of infection with severe acute respiratory syndrome coronavirus 2 (SARS-CoV-2) involves the angiotensin-converting enzyme 2 (ACE2) located on the cell surface in initiating the viral entry process [26, 27]. During infection, the receptor-binding domains (RBDs) of the S1 protein recognize and bind to the ACE2 receptor on the cell surface [28]. After intracellular replication, the SARS-CoV-2 coronavirus, particularly in obese patients with excessive visceral fat accumulation, hyperactivates cellular and humoral immune pathways, leading to excessive inflammatory reactivation and potentially a “cytokine storm,” which can cause acute respiratory distress syndrome (ARDS), massive bilateral pneumonia, kidney, liver, and heart damage, and consequently, the development of multi-organ failure [29]. On the other hand, comorbidities may influence the severity and mortality of SARS-CoV-2 infection. Significant differences in both severity and mortality were observed between control groups and groups with type 2 diabetes and obesity, as confirmed by various studies [30]. Differences were also observed among ICU (intensive care unit) patients. Before the pandemic, obese patients accounted for 15–20% of all admissions. The pandemic caused a two-fold increase (to 40%) among these individuals [22]. This indicates that both conditions are strong predictors of prognosis after COVID-19 infection (Table 2). A cohort study by Zhou et al. found that 31% of people who died from COVID-19 in China had diabetes [23].

## SARS-CoV-2 virus variants

Individual SARS-CoV-2 variants differ in their cell penetration characteristics and protein characteristics. The Omicron variant (Ο) is characterized by a greater affinity for ACE2 receptors than the previous subtypes. Considering the overexpression of this receptor in the group of patients with diabetes and obesity, it predisposes them to easier infection and more severe course of disease [31]. Effect of ACE2 polymorphism on mortality was examined in the Omicron BA.5, Alpha and Delta groups by Sheikhian F et al. [32]. ACE2 rs2074192 TT allele correlated with higher mortality in patients in all groups. Additionally, ACE2 rs2074192 CT was associated with higher mortality in the case of Omicron BA.5 and Delta subtypes. ACE2 rs2074192 TC genotype with higher mortality of Omicron BA.5 and Alpha variants and TT genotype in Delta groups [32]. This illustrates the variability between individual mutations of the SARS-CoV-2 virus and the importance of the role of the ACE2 receptor in the course of the disease. SARS-CoV-2 infection changed dynamically over time with the emergence of new mutations of the virus. Understanding the characteristics of a given variant translated into the diagnosis and treatment of the disease. The variability mainly concerned the RBD fragment responsible for binding to the human ACE2 receptor.

## Diabetes as a risk factor in COVID-19

Excessive fat accumulation leads to increased release of IL-6 and TNF-α, followed by activation of macrophages and development of inflammation inducing decreased insulin sensitivity. This leads to disruption of lipolysis processes and increased production of free fatty acids (FFA), resulting in decreased glucose utilization. In addition, glucose uptake by muscle and adipose tissue is impaired, and glucose production in the liver is increased [33, 34].

**Fig. 3:**
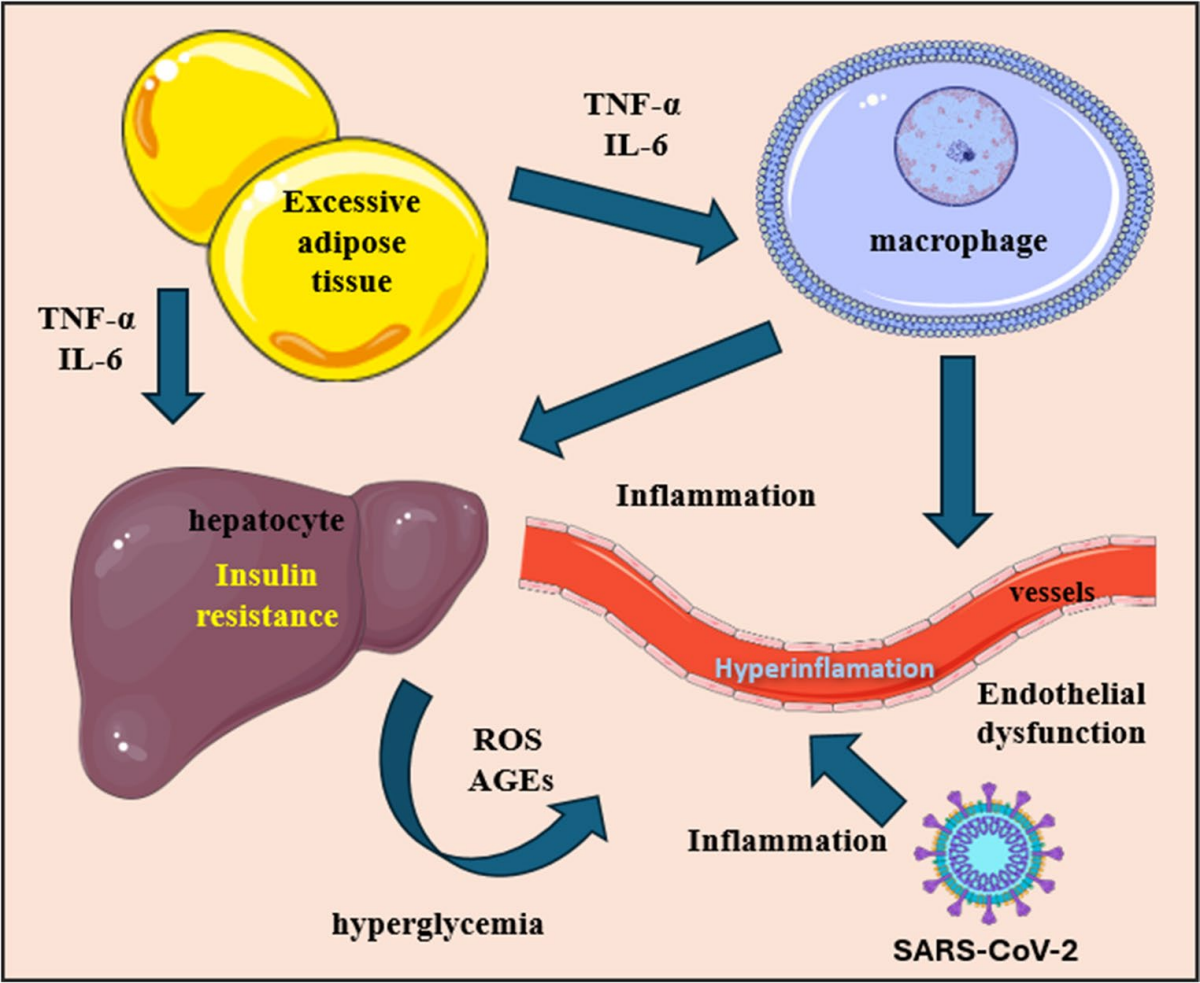
Simplified visualisation of the relationship between inflammation generated by excessive accumulation of adipose tissue and SARS-CoV-2 infection on glucose metabolism and vascular endothelium. Excessive adipose tissue secretes TNF-α and Il-6 which both mediates macrophages activation and hyperglycemic response in liver cells [108, 109]. In terms of virus infection pre-existing inflammation combines with the one mediated by immune response. It creates hyperinflammation which affects vessel and leads to endothelial dysfunction [50, 51]. Figure 3 was created using the Servier Medical Art Commons Attribution 4.0 Unported Licence http://smart.servier.com. https://creativecommons.org/licenses/by/4.0/

As the COVID-19 pandemic progresses, diabetes and metabolic disorders have become the dominant risk factors, replacing the previously perceived chronic obstructive pulmonary disease (COPD) [41]. Patients with diabetes are more likely to require hospitalization and present with a more severe course of infection. The incidence of major symptoms (Table 1), as well as the development of multi-organ damage and mortality, is increasing in this group [42]. Oxidative damage induced by hyperglycemia also contributes to the progression of vascular structure and function disorders (Fig. 3) [43]. Severe infectious diseases superimposed on existing vascular diseases increase the risk of thrombotic and ischemic events [44]. Thromboembolic complications were frequently observed in both patients with SARS-CoV infection admitted to the ICU and those suffering from long-term COVID [45].

**Table 1:** Comparison of selected symptoms of hospitalised COVID-19 patients with (T2DM) or without (C) pre-existing diabetes mellitus type 2 [35–40]

	C	T2DM	C	T2DM	C	T2DM	C	T2DM	C	T2DM
**Published data**	15.08.2023		25.01.2021	7.08.2023	10.07.2020		26.10.2020		08.2020	
**Cough**	60.8%	72.7%	45.5%	64.1%	83.1%	89.3%	62.1%	59.7%	65.6%	71.4%
**Fever**	82.8%	85.9%	50.6%	83,70%	81.9%	89.3%	78.9%	85.1%	83.1%	79.4%
**Dyspnea**	39.2%	66.2%	-	22.2%	33.1%	66.7%	24.7%	20.9%	47.7%	49.2%
**Muscle ache**	28.2%	29.5%	1.3%	18.1%	24.1%	20.2%	9.1%	13.4%	14.4%	19.0%
**Diarrhoea**	5.3%	6.2%	1.3%	33.2%	20.5%	32.1%	9.0%	10.9%	19.5%	27.0%
**Vomiting**	7.4%	9.4%	-	5.5%	-	-	2.3%	4.5%	6.2%	9.5%
**Nausea**	2.6%	2.6%	-	-	-	-	4.2%	9.0%	13.3%	7,9%
**Tightness in chest**	4.5%	5.3%	17.9%	2.9%	40.4%	54.8%	23.7%	19.4%	4.7%	3.2%
**Headache**	11.6%	5.8%	3.2%	-	13.9%	9.5%	3%	3%	12.8%	4.8%
**Stomachea**	7.2%	2.9%	-	3.5%	-	-	0.8%	0%	-	-
**Tiredness**	18.2%	22.1%	14.7%	-	58.4%	63.1%	30.5%	38.8%	29.5%	34.9%
**Poor appetite**	3.4%	4.6%	8.3%	-	51.2%	76.2%	-	-	-	-
***Ref***	*35*	*35*	*36*	*37*	*38*	*38*	*39*	*39*	*40*	*40*

C – control group of COVID-19 patients, T2DM – T2DM group of COVID-19 patients

The production of reactive oxygen species (ROS) and other free oxygen radicals in the mitochondria of endothelial cells is also increased in obese patients infected with the SARS-CoV-2 virus [13]. Glutathione (GSH) deficiency, reduced synthesis of superoxide dismutase (SOD) observed in patients with diabetes [14], as well as catalase (CAT) and peroxidase in diabetic rat models contribute to the redox imbalance [46]. A study showed a twofold lower concentration of GSH in human red blood cells from diabetic patients compared to healthy individuals. Reduced concentrations were also observed in blood plasma [47, 48]. Studies of HUVEC cells treated with protein S showed higher ROS generation in the presence of high-glucose medium in vitro, indicating that hyperglycemia may contribute to increased oxidative imbalance during SARS-CoV-2 infection [49]. High levels of ROS, which influence NO production in the vasculature, cause lipid peroxidation and, together, induce the development of atherosclerosis, characteristic of patients with diabetes (Fig. 3) [50]. It also activates pro-inflammatory pathways causing chronic inflammation, which may promote the development of severe infection in the course of COVID-19 [51]. The immune response itself may also be modulated by chronic hyperglycemia. This condition is associated with impaired innate and adaptive immunity, impaired cytokine response, neutrophil abnormalities and slowed leukocyte recruitment [52]. This is confirmed by the study conducted by Guo et al. [53], who showed reduced lymphocyte counts and higher absolute neutrophil counts. Higher levels of IL-6, ESR, ferritin and CRP were observed in the population of patients with COVID-19 and diabetes [53]. An improperly functioning immune system also increases the risk of secondary bacterial, viral and fungal infections in the course of SARS-CoV-2 infection [54].

Hyperglycemia associated with diabetes causes thickening of the alveoli, epithelium, and basal lamina, which adversely affects the gas exchange process [55]. Through non-enzymatic glycation, collagen becomes more resistant to proteolysis and, as a consequence, restrictive lung disease develops [56]. Mucociliary clearance is also impaired, which impairs the clearance of pathogens present in the mucus and allows their multiplication [57]. It may also be accompanied by respiratory muscle weakness [55–57], reducing the initial diffusion capacity, forced vital capacity (FVC), and forced expiratory volume in 1 s [58]. Diabetes also alters the abundance of some proteins in lung tissues. Wijnant et al. (2020) reported higher ACE2 protein levels, but not mRNA synthesis, in alveolar tissue and bronchial epithelium of diabetic patients [15]. Higher expression of ACE2 in the lungs has also been observed after severe COVID-19, as evidenced by protein expression analyses of *post-mortem* lung tissue [17]. A state of persistent hyperglycemia may lead to overexpression of ACE2 and its hyperglycosylation [16, 59], which may have an impact on the progression of COVID-19 and its association with diabetes [60]. Although several other candidate receptors have been proposed, ACE2 seems to be of major importance in SARS-CoV-2 infection in vivo [61].

The difference in mortality due to COVID-19 was observed in diabetic patients with controlled and uncontrolled glucose levels. The mortality rate due to diabetes in COVID-19 was 28%, compared with 6.2% in the control group (Table 2). Uncontrolled hyperglycemia, exceeding 180 mg/dL, resulted in a mortality of 41.7%, while only 14.8% of patients with lower glucose levels died [66]. One of the acute conditions associated with diabetes and uncontrolled glucose levels is diabetic ketoacidosis (DKA). Its episode can occur during SARS-CoV-2 infection. A study conducted in Wuhan, China, showed that 7% of patients hospitalized with COVID-19 developed ketonemia or ketonuria, which allows the diagnosis of DKA. A common feature of all patients in this group was pre-existing diabetes [67]. Other studies documented the occurrence of DKA in 2% of patients hospitalized with COVID-19 and a 50% mortality rate in the case of its development during infection [18]. Table 3 shows the main diagnostic parameters that differ in COVID-19 patients with or without type 2 diabetes. A characteristic phenomenon of SARS-CoV-2 infection is a low level of lymphocytes and neutrophils compared to healthy individuals. At the same time, it has also been suggested that an increased level of cells may indicate disease progression [68]. Higher white blood cell (WBC) and neutrophil counts have been observed in COVID-19 patients with pre-existing diabetes (Table 3). Disorders related to the coagulation system are also associated with diabetes [69]. A decrease in fibrinolytic activity and an increase in prothrombotic activity are observed, which is represented by an increased level of D-dimer [70]. Meta-analyses provided by Varikasuvu et al. (2021) [71] indicate the importance of this diagnostic parameter in predicting the progression of COVID-19. Increased plasma D-dimer levels were found to be associated with both increased symptom severity and mortality. Ferritin has been suggested as a marker of lung injury in COVID-19, but no significant correlation was found with intensive care unit (ICU) admission or mortality [72]. Later studies also indicate this association [73, 74], although iron and transferrin have been suggested as candidates for better prognostic biomarkers for COVID-19. Other factors differentiating COVID-19 patients without diabetes and with diabetes were creatinine, aspartate aminotransferase (AST) and lactate dehydrogenase (LDH) (Table 3). It has been suggested that AST should be considered a clinical marker of risk of mortality due to COVID-19 [75]. Increased plasma AST activity was observed in ICU patients and ICU-COVID-19-nonsurviving patients. It has been suggested that this phenomenon may be related to hypoxia caused by respiratory infection, as previously shown in H1N1 influenza infection [76]. Elevated LDH and creatinine levels have also been suggested as risk factors for severe COVID-19 or mortality, as in the case of creatinine [77]. Increased mortality was associated with the occurrence of acute kidney injury (AKI) [78]. On the other hand, COVID-19 infection may cause metabolic disorders that seem to be the result of increased cytokines and inflammatory mediators circulating in the blood. As a consequence, insulin resistance develops and concomitant hyperglycemia, which is also an isolated risk factor in the course of SARS-CoV-2 infection [79]. Its acute course is possible. Considering the dynamics of this process, blood glucose concentration has a greater diagnostic value than HbA1c [44]. A study conducted by Zhou et al. showed glycemia disorders in 69% of patients [80]. Treatment itself in the course of infection may also induce hyperglycemia. The problem of hyperglycemia induced by the use of glucocorticosteroids is often overlooked. However, considering the poorer prognosis in patients who have experienced it, its development should be prevented and if it occurs, it should be appropriately corrected [81].

**Table 2:** Mortality and severity of SARS-CoV-2 infection in patients with type 2 diabetes or obesity compared to the control groups [35–40, 62–65]

	T2DM	C	T2DM	C	T2DM	C	T2DM	C	T2DM	C	T2DM	C	T2DM	C	Obesity	C	Obesity	C	Obesity	C
**Total (n)**	427	656	31	-	343	-	84	312	67	596	63	195	25	108	123	203	46	38	41,344	110,987
**Severe COVID**	42 (9.8%)*	15 (2.3%)*	-	-	56** (16.3%)	-	3 (3.6%)*	6 (1.9%)*	53 (79.1%)***	356 (59.7%)***	45 (71.4%)***	126 (64.6%)***	5 (20.0%)*	9 (8.3%)*	36 (29.3%)	39 (19.2%)	16 (34.8%)*	7 (18.4%)*	1766 (4.27%)*	1773 (1.59%)*
**Mortality**	63 (14.8%)	32 (4.9%)	20 (64.5%)	-	-	-	-	-	3 (4.5%)	22 (3.7%)	7 (11.1%)	8 (4.1%)	10 (9.3%)	4 (3.7%)	1 (0.8%)	1 (0.5%)	16 (34.8)	12 (31.6%)	6481 (15.5%)	7135 (6.42%)
**Ref.**	35		36		37		38		39		40		62		63		64		65	

C – control group of COVID-19 patients, T2DM – T2DM group of COVID-19 patients, *Defined as application of intubation (mechanical ventilation), ** Defined as need of hospitalisation, *** Defined by other criteria

**Table 3:** Diagnostic and demographic parameters of COVID-19 patients with (T2DM) or without (C) pre-existing diabetes mellitus type 2 [35, 38, 39, 82]

	C	T2DM	C	T2DM	C	T2DM	C	T2DM
**Male/Female** **n (%)**	51.3/48.7	60.3/39.7	78/22	88.5/11.5	-	59.5/40.5	53.7/46.3	60/40
**Age**	64 (55–70)	65 (57–71)	-	-	58.5 (49–67)	62 (55–70)	48 (33–61)	63 (52–72)
**WBC** **(x10⁹/l)**	5.45 (4.31–7.19)	6.34 (4.66–8.15)	8.31	9.62	5.88 (4.51–6.43)	-	-	-
**Neutrophils (x10⁹/l)**	3.82 (2.81–5.39)	4.49 (3.12–6.91)	-	-	-	3.6 (2.4–5.7)	3.87 (2.70–5.23)	4.27 (2.41–6.83)
**Lymphocytes (x10⁹/l)**	0.995 (0.72–1.37)	1.1 (0.65–1.41)	-	-	-	0.8 (0.6–1.2)	1.54 (1.01–2.12)	1.26 (0.87–1.73)
**CRP** **(mg/l)**	30.68 (5.75–67.37)	30.75 (4.53–81.72)	50.23	86.38	5.53 (2.08–18.2)	28.1 (13.0–76.7)	5.80 (2–47)	28 (8-173)
**LDH** **(IU/l)**	264.5 (207–338)	318 (195.5-426.3)	355.55	444.93	-	-	-	-
**Ferritin** **(µg/l)**	-	-	1231.68	1526.7	210 (114.7–487.0)	-	140 (41–302)	501 (153–1102)
**ALT** **(IU/l)**	-	-	-	-	34 (20.0–52.5)	28 (19–39)	19 (14–33)	27 (19–39)
**AST** **(IU/l)**	-	-	-	-	25.0 (17.5–43.0)	34 (20–47)	23 (18–31)	30.00 (21–41)
**D-dimer (mg/l)**	0.54 (0.25–1.51)	0.87 (0.35–2.46)	2.46	4.14	-	0.78 (0.34–1.38)	4.44 (2.75–8.77)	6.65 (5.64–19.27)
**Creatinine (µmol/l)**	67.8 (57.2-78.23)	74 (64.25–95.78)	102.5	165.3	66.3 (58.9–87.2)	-	-	-
***Ref***	*39 39*		*35 35*		*38*	*38*	*82 82*	

C – control group of COVID-19 patients, T2DM – T2DM group of COVID-19 patients; WBC, white blood cell; CRP, C-reactive protein; LDH, lactate dehydrogenase; ALT, alanine transaminase; AST, aspartate transaminase

In terms of pharmacotherapy, metformin is the recommended drug. It can be used in patients with asymptomatic or mild symptoms of COVID-19. However, it poses a risk of lactic acidosis, especially in patients with severe coronavirus disease [62]. Acarbose is an inhibitor of α-glucosidase in the jejunal epithelium. It has been evaluated as monotherapy and in combination with metformin. In both cases, it resulted in a higher survival rate in patients with COVID-19 [83]. Clinical trials of the combination of pamapimod and pioglitazone, which initially revealed antiviral potential by inhibiting SARS-CoV-2 replication, were stopped due to a small impact on clinical outcomes [84]. An isolated effect of pioglitazone on reducing IL-3 levels in patients has been documented without affecting other inflammatory markers compared to placebo. However, patients from the study group were more often admitted to the ICU [85]. The use of sulfonylureas is not recommended because they can cause hypoglycemia [86]. The adverse effects of GLP-1 agonists include nausea and vomiting. This can result in dehydration and aspiration pneumonia. For this reason, they should not be used in patients with severe COVID-19 [86]. However, in the study conducted by Benjamin M. Scirica et al., a reduced mortality due to infectious causes was observed in the group treated with Semaglutide during the Pandemic [87]. Due to the low risk of hypoglycemia and amelioration of the course of infection, it is possible to continue treatment with DPP-4 inhibitors [88]. Therapy with SGLT-2 inhibitors may also have positive effects. They are believed to have strong antiviral effects and reduce the level of IL-6 accompanying infection [89]. The method of choice in the treatment of hospitalized patients is intravenous insulin administration. This approach allows for precise glycemia control and has additional anti-inflammatory effects. The issue of vaccines is controversial. Li H et al. [90] conducted a cross-sectional study in China, which included mainly patients with diabetes (87.7%). They noted that 6% of people after both the first and second dose of the vaccine reported adverse events. The most common were fatigue, muscle pain, and insomnia. These results translated into the number of people vaccinated with subsequent doses: 16.2% received two doses, 65.2% - three. The final results did not show any serious complications. Another study raises a different issue, namely the quality of the immune response in patients with T2DM. Yuan S et al. [91] noted that such people developed a weaker immune system reactivity in response to SARS-CoV-2 virus vaccines. This was associated with lower production of specific IgG anti-SARS-CoV-2 antibodies and, consequently, with poorer prevention of COVID-19 infection. Another issue of adverse effects is the occurrence of *de novo* diabetes in previously healthy patients [92]. There are also cases of activation of diabetes from prediabetes [93] or transformation of T2DM into T1DM [94]. Almost all patients with such side effects were effectively treated with insulin, ultimately achieving optimal glycemic control. Most studies refer to post-vaccination complications and negative attitudes of people with diabetes, but positive effects of vaccination, improved outcomes, and easier survival of COVID-19 have been proven [95]. Guoxi Cai et al. [96] demonstrated a beneficial effect of vaccination in particularly vulnerable individuals, including obese patients. Full vaccination was associated with a lower risk of hospitalization and severe disease. Abdominal obesity, a risk factor in COVID-19 infection, was associated with a higher amount of IgG anti-SARS-CoV-2 antibodies in patients after the COVID-19 disease and after vaccination. However, they had lower avidity. The study included 5 vaccines: AZD1222, Convidecia, BNT162b2, Sputnik V, and CoronaVac. Abdominal obesity has not been shown to affect the reactivity of these vaccines [97]. Another study showed a similar risk of disease after vaccination in obese and non-obese patients. Vaccination resulted in comparable immunogenicity [98]. The effect of vaccination in a group of people with normal body weight, overweight and obesity was examined in the context of sleep disorders and the chance of developing long COVID. In people with obesity, the chance of developing long COVID was increased compared to the other groups. The authors linked this to dysfunctions such as insomnia and obstructive sleep apnea and the role of sleep on the functioning of the immune system [99]. The impact of obesity on vaccination against SARS-CoV-2 is not fully understood. The results differ between authors. However, as obesity is one of the factors of severe COVID, vaccination is recommended in this group of people.

## Obesity and COVID-19

Many sources link obesity with increased mortality in the course of COVID-19 infection. Studies conducted in several Chinese hospitals, including the Third People’s Hospital in Shenzhen, focused on people with overweight (*BMI* from 25.0 to 29.99 kg/m 2 ) or obesity (*BMI* ≥ 30.0 kg/m 2 ) and co-infection with SARS-CoV-2, showed a higher incidence of severe pneumonia and, consequently, the need to implement oxygen therapy in a medical facility, than in the group with normal body weight (*BMI* from 18.5 to 24.99 kg/m 2 ) [63]. A meta-analysis was also performed [49]. It proved that patients with *BMI* ≥ 30 kg/m2 had a 1.33-fold higher and those with *BMI* ≥ 40 kg/m2 had a 4.6-fold higher risk of pneumonia compared to normal-weight patients with COVID-19. People with severe obesity (*BMI* ≥ 40 kg/m2 ) showed, independent of other factors, worse outcomes in medical facilities and higher in-hospital mortality [64]. Similar results were reported in a large cohort study from Mexico [65]. Obesity was shown to increase the risk of pneumonia in COVID-19 by 1.327-fold [100]; the risk of hospital admission was also increased by 1.29-fold [65]. Further evidence was provided by data from the US Centers for Disease Control and Prevention, which studied patients infected with the SARS-CoV-2 coronavirus. It was reported that 69% of them had a *BMI* in the range of 30–40 kg/m 2, while 30.1% had a weight ≥ 40 kg/m 2 [101]. Lighter et al. showed that people with a *BMI* in the range of 30–35 kg/m 2 were admitted to the ICU 1.8 times more often, and those with a *BMI* above 35 kg/m 2–3.6 times more often, compared to patients with a *BMI* not exceeding 30 kg/m 2 [102]. Excess body weight has an adverse effect on the respiratory system, its mechanics, physiology and anatomy [103]. It leads to weakening and worse functioning of the respiratory muscles, and thus worse gas exchange and reduced lung volume. The SARS-CoV-2 coronavirus infects the human body mainly through droplets inhaled into the respiratory system, which leads to the release of cytokines and worse function of the lung alveoli. Obese individuals infected with COVID-19 have a significantly higher risk of respiratory complications. This is associated with a poorer prognosis, a higher probability of a severe course [104] and a more frequent need for mechanical ventilation and oxygen therapy [105]. An animal model study showed the development of more severe inflammation in the lungs in mice fed a fatty Western diet equivalent compared to standard chow. They also had increased markers of inflammation and overexpression of genes of pathways associated with inflammatory pathways [106]. In the obese group, a 5% increased risk of ARDS was found compared to the group with normal body weight [107].

This is due to, among other things, chronic inflammation in the visceral adipose tissue. Adipocytes produce large amounts of pro-inflammatory cytokines and contain ACE2 receptors on their surface. The coronavirus SARS-CoV-2 can penetrate into adipose tissue by binding to these receptors, as demonstrated by Ryan and Caplice [108]. They also noted that increased levels of IL-6 in obese people in the case of COVID-19 infection can lead to a “cytokine storm”, which leads to significant organ damage and wasting. Similar studies were conducted by Misumi et al. [110] in a mouse model. The aim was to demonstrate the overexpression of virus-specific memory T cells in the spleen and adipose tissue of obese people. They found that high levels of IL-6 and a large number of ACE-2 receptors in adipocytes may be associated with an unfavorable prognosis and higher severity of COVID-19 in obese patients. Furthermore, in obese individuals, immunocompetent cells are resistant to leptin signaling [111]. This causes negative effects on the production and development of T lymphocytes and an impaired immune response. Adipose tissue is also deposited in the epicardial area and is called EAT (epicardial adipose tissue). It is a significant source of adipokines and proinflammatory mediators, which promotes the formation of a “cytokine storm” [112]. Obese individuals are characterized by impaired angiotensin 1–7 signaling, resulting in chronic activation of the RAAS and high expression of ACE2, which affects the antiviral immune response and facilitates viral entry [109]. However, angiotensin II and angiotensinogen levels increase, leading to lung damage, inflammation, fibrosis, and in extreme cases, ARDS [113, 114]. Excess adipose tissue is also associated with excessive complement activation associated with more severe inflammation [115]. Another aspect of obesity is the frequently observed vitamin D3 deficiency [116]. This may result in more frequent lung infections, but also inflammation, respiratory diseases, systemic infections, and impaired immune response [117]. Due to the above-mentioned mechanisms, obese people are at risk of more severe viral infections due to impaired immune response, chronic inflammation, and limited lung function [118]. Obese people often have comorbidities such as hypercholesterolemia and hypertension. Pharmacotherapy for these conditions may also affect the treatment of SARS-CoV-2. Continuation and initiation of statin therapy during COVID-19 infection may be beneficial for patients. In addition to their effects on lipid metabolism, statins reduce oxidative stress, have antithrombotic and immunomodulatory effects [119]. As a result, they reduce the cytokine storm by reducing the production of cytokines and inflammatory markers [120]. Statin use has been shown to be associated with significantly reduced mortality and a lower risk of ICU admission in COVID-19 patients [121]. Questions have been raised about the potential impact of angiotensin-converting enzyme inhibitors (ACEIs) and angiotensin receptor blockers (ARBs) on the course and risk of COVID-19 infection. Inhibition of ACE2 by ACEIs results in a decrease in angiotensin I levels, which consequently leads to increased ACE2 expression. Increased receptor numbers expose individuals taking ACEIs to SARS-CoV-2 infection [122]. The results of a study by Zhang et al. [123] do not indicate an additional benefit of ACEIs and ARBs in terms of higher survival compared to patients taking statins alone. However, ACEIs may also reduce systemic inflammation. In the hospital setting, continuation of ACEI/ARB treatment in patients previously taking them on an outpatient basis resulted in a reduced mortality compared to the group in which these drugs were discontinued [124]. Recommendations have been made to continue ACE inhibitors and ARBs in patients who have already received them for COVID-19 [125]. The UK study, RECOVERY, showed a positive effect of dexamethasone in patients with severe SARS-CoV-2 who required mechanical ventilation or oxygen therapy [126, 127]. The results showed reduced mortality, which led to changes in WHO recommendations and a beneficial effect of steroids in the treatment of COVID-19 [128].

Regarding vaccination against SARS-CoV-2 virus in obese people, a study was conducted showing a correlation between body weight and IgG anti-SARS-CoV-2 antibodies. Ou X et al. [129] suggest that with increasing BMI, the humoral response of the body decreases. The level of IgG antibodies produced in the obese group is lower and lasts shorter. These conclusions were also drawn by Fu C et al. [130]. In addition, the researchers noted that non-obese people reported more adverse effects after vaccination. However, these studies have the same conclusions. Despite a weaker humoral response, obese people had an easier time with COVID-19 after vaccination [131]. The analysis we recently conducted showed that body mass composition and anthropometric parameters can affect immune response after vaccination [132]. We found a negative correlation between body fat mass and IgG titer against SARS-CoV-2. The same study showed that higher muscle mass is a factor for higher IgG titer after vaccination [132]. This suggests that body composition may influence vaccination. Obesity may negatively affect its efficacy and result in lower protection against infection.

### Cytokine storm

**Fig. 4:**
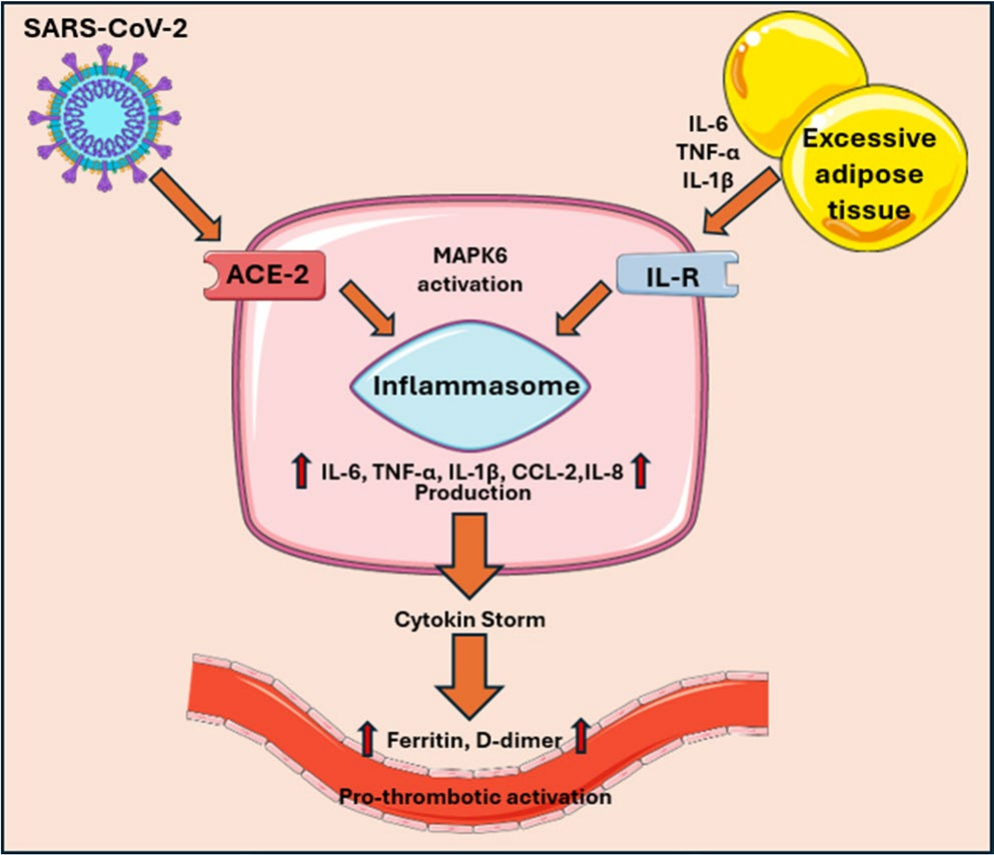
Excessive adipose tissue and SARS-Cov-2 infection influence cytokine storm course and its prothrombotic effect on vessel endothelium. Excessive adipose tissue through Il-R and Sars-CoV-2 virus through ACE-2 affects MAPK6 activation. It leads to formation of inflammasome [139, 140]. Pro-inflammatory cytokine production is upregulated, and in this terms cytokine storm is initiated [133]. Circulating cytokines damages endothelium and lifts thrombotic risk. It can be observed in higher serum levels of Ferritin and D-dimer [135–138]. Figure 4 was created using the Servier Medical Art Commons Attribution 4.0 Unported Licence http://smart.servier.com. https://creativecommons.org/licenses/by/4.0/

The “cytokine storm” involves the activation of numerous leukocytes, endothelial cells, mast cells, and macrophages. A large number of proinflammatory cytokines and chemokines are released from the cells [133]. The pathogenesis of this process is particularly associated with the participation of mast cells in the inflammatory response, via TLRs [134]. These cells also have the ability to recognize DAMPs (damage-associated molecular patterns). This allows them to detect and bind to the SARS-CoV-2 virus [135–138]. Coronavirus infection is associated with prolonged prothrombin time, increased levels of D-dimers, and procoagulant factors (Fig. 4). This may result in a worsening of the patient’s condition, prognosis, and increased mortality [135–138].

### The risk of death and severe course of COVID-19 in obese patients with diabetes type 2

**Fig. 5:**
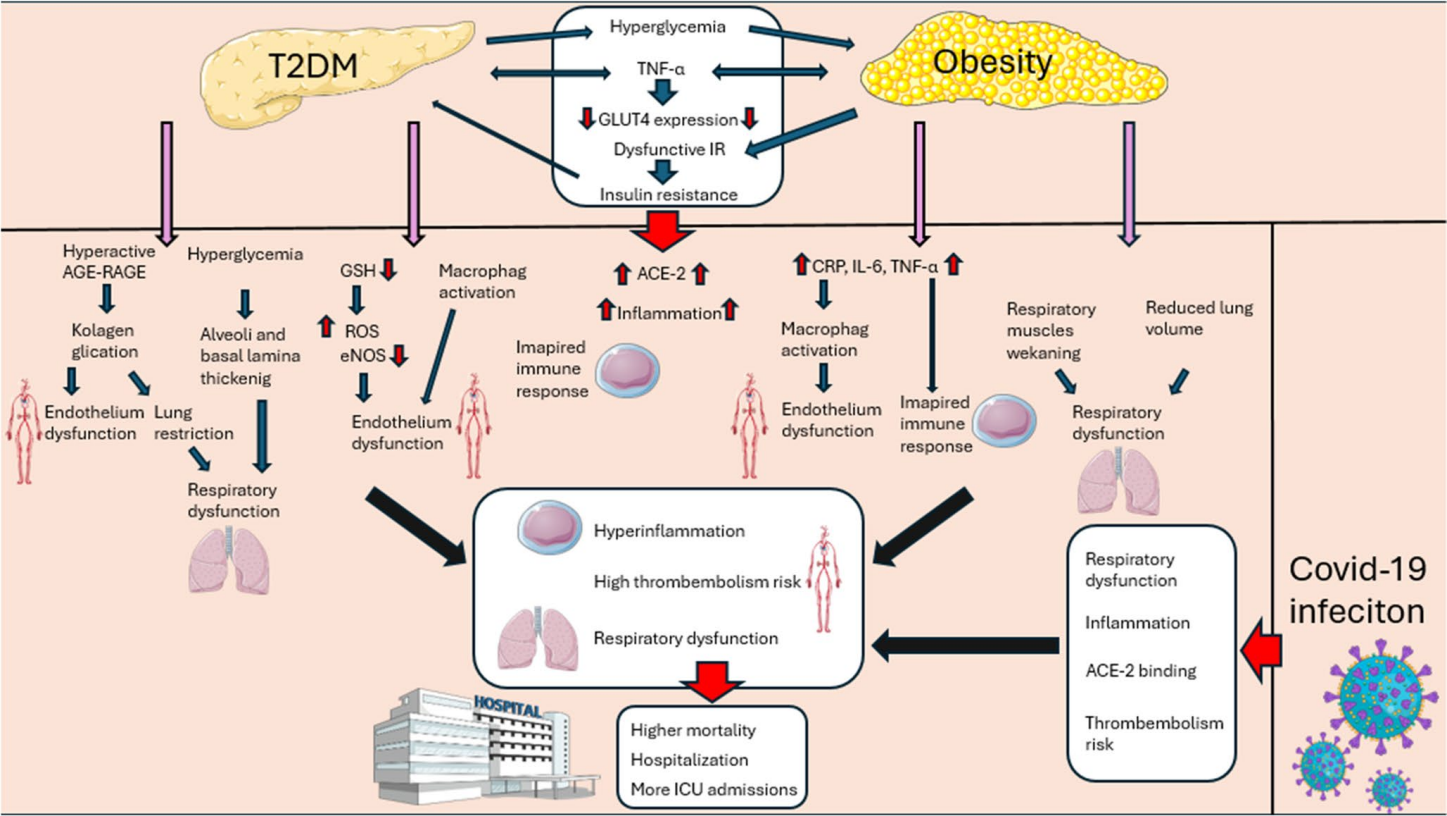
The links between T2DM and obesity and their impact on viral infections are multi-stage. Both obesity is a factor in the development of diabetes *via* TNF-α signaling, decreased expression of the GLUT4 receptor, and insulin resistance [9, 33, 34]. T2DM itself also promotes abnormal body weight through hyperglycemia and elevated TNF-α levels. There is also overexpression and glycation of ACE2, which is the route of entry of the SARS-CoV-2 virus into cells [16, 28, 59]. In the presence of comorbidity, many unfavorable phenomena occur that affect the risk of severe infection. The immune, respiratory, and vascular systems are disturbed *via* interleukin signaling, protein glycation, and decreased antioxidant capacity [13, 14, 43, 52, 56, 69, 103, 108]. In an infection such as COVID-19, respiratory failure, increased risk of thromboembolism and inflammation may occur. The presence of these disorders predisposes this group of patients to a severe course of infection, increases the likelihood of implementing intensive care and results in higher mortality [18, 41, 64, 66, 104, 105]. Figure 5 was created using the Servier Medical Art Commons Attribution 4.0 Unported license http://smart.servier.com. https://creativecommons.org/licenses/by/4.0/

Diabetes and obesity are documented isolated risk factors for severe COVID-19. It has been shown that as many as 87.5% of the adult population suffering from T2DM are overweight [141]. Severe COVID-19 is largely associated with excessive activity of pro-inflammatory factors (cytokine storm). Its occurrence increases the risk of death by 15–20% [43]. Obesity and diabetes are chronic diseases that increase inflammation affecting the physiological balance of the body. Existing, long-term, mild inflammation is the basis for the development of inflammatory hyperactivation in the case of favorable factors, such as viral infection [142]. Subsequent activation and dysfunction of endothelial cells leads to increased thrombotic risk [45]. Diabetes and obesity also affect the immune response. People with these diseases are more susceptible to infections and acute conditions accompanying them. The co-occurrence of diabetes and obesity increases the risk of COVID-19 progression by 4.5-fold, while in people with one of these diseases the risk is 3.5-fold higher than in the general population [143]. It was observed that a *BMI* of 35 kg/m2 or higher was associated with a more severe course of COVID-19 infection and an increased risk of invasive mechanical ventilation, mainly due to visceral adipose tissue. In the report of Ogata et al. (2021) [144], taking into account the severity of SARS-CoV-2 infection, they revealed that the ratio of visceral to total adipose tissue is an independent risk factor, and not *BMI* or even visceral adipose tissue itself, the same one that contributes to the development of insulin resistance, discussed by Dhokte and Czaja (2024) [145]. Similar observations were reported by Saccon et al. (2022) [146]. This was associated with a higher rate of proinflammatory cytokine synthesis in visceral adipocytes compared to subcutaneous adipose tissue, which together may be one of the links between diabetes, obesity, and COVID-19 severity, as visceral adipose adipocytes express higher levels of ACE2 and are more susceptible to SARS-CoV-2 infection [146]. Nevertheless, *BMI* remains a useful tool for risk assessment until adipose tissue distribution is also taken into account [147] (Fig. 5).

## Limitations of the study

This review has its limitations. During the selection process, one of the criteria was that the text had to be in English, works in other languages were not considered. Another limitation is the heterogeneity of the source studies. The diversity of the populations, their geographic distribution, and therefore demographic differences. These factors may vary depending on the phase of the pandemic or the healthcare system. It is also important to note the risk of bias in the studies selected for review by the authors. It was impossible to include all possible sources of information due to their sheer number. This was done in order to maintain the readability and clarity of the text. The manuscript includes works that the authors subjectively considered to be the most informative and unique. However, the greatest limitation is time and the rapidly evolving knowledge of available information, which may result in the presented results becoming rapidly outdated.

## Conclusions

Obesity and diabetes affect the severity and mortality of COVID-19. The cause of the phenomenon is multidimensional. The development and progression of type 2 diabetes are usually associated with obesity, especially with excess visceral fat, which triggers many events such as cytokine imbalance [148], chronic inflammation [149] or susceptibility to oxidative stress [51], affecting insulin signaling and glucose or lipid metabolism. Uncontrolled hyperglycemia is particularly dangerous and can lead to ketoacidosis and the development of many comorbidities characteristic of diabetes and obesity. SARS-CoV-2 seems to use these events to induce a more effective infection, triggering damage to multiple organs and, in the acute stage, a hyperinflammatory immune response, resulting in a more severe course of COVID-19 and increased mortality. Further studies are needed to clarify which of the specific events observed during the progression of infection are most important and determine the severity of COVID-19. This can help prevent COVID-19 from becoming severe, but it can also be beneficial in understanding the mechanism of viral infection in general. But the actions should be broader, concerning the introduction of new treatment patterns, as well as new methods of prevention. In addition, new guidelines for managing the global epidemic state should be developed for the future, based on the experience already gained during the COVID-19 pandemic.

## Key references

- Alawadi F, Bashier A, Bin Hussain AA, Al-Hashmi N, Bachet FAT, Hassanein MMA, et al. Risk and predictors of severity and mortality in patients with type 2 diabetes and COVID-19 in Dubai. World J Diabetes. 2023;14:1259–70.
  - This cross-sectional, nested case-control study assessed risk-adjusted predictors of severity and mortality among patients hospitalised with COVID-19 and comorbid type 2 diabetes. The study was conducted during the first wave of the pandemic in Dubai.
- El Feky AY, Abdrabo MG, Abdou MS, Sabry SH, Badr AA, Mohsen AM. Factors Affecting Outcomes of COVID-19 Infection among Older Adults with Type 2 Diabetes: A Single Center, Cross-Sectional Study. Clinical Diabetology. 2023;12.
  - A retrospective case series study aimed at analysing laboratory, radiological and clinical results among elderly patients hospitalised with COVID-19 and suffering from type 2 diabetes.
- Obiri-Yeboah D, Bena J, Alwakeel M, Buehler L, Makin V, Zhou K, et al. Association of Metformin, Dipeptidyl Dipeptidase-4 Inhibitors, and Insulin with Coronavirus Disease 2019–Related Hospital Outcomes in Patients with Type 2 Diabetes. Endocrine Practice. 2023;29.
  - Retrospective study of patients treated in a single hospital system using insulin, dipeptidyl dipeptidase-4 inhibitors and metformin due to COVID-19 with comorbid type 2 diabetes.
- Valencia I, Lumpuy-Castillo J, Magalhaes G, Sánchez-Ferrer CF, Lorenzo Ó, Peiró C. Mechanisms of endothelial activation, hypercoagulation and thrombosis in COVID-19: a link with diabetes mellitus. Cardiovasc Diabetol. 2024.
  - This review presents the mechanisms of thrombosis, hypercoagulation and endothelial activation in patients infected with the SARS-CoV-2 virus with concomitant diabetes. This is a comprehensive review analysing numerous studies on this topic. It constitutes an important summary of knowledge on this topic.
- Dhokte S, Czaja K. Visceral Adipose Tissue: The Hidden Culprit for Type 2 Diabetes. Nutrients. 2024;16:1015.
  - This article reviews information about body fat as a major determinant leading to the development of type 2 diabetes.

## Data Availability

No datasets were generated or analysed during the current study.

## Declarations

Figures 1, 3, 4 and 5 were created using the Servier Medical Art Commons Attribution 4.0 Unported Licence https://smart.servier.com. https://creativecommons.org/licenses/by/4.0/.
